# 599. Patient Beliefs Regarding Lyme Disease and Need for Antimicrobial Treatment when Referred for Lyme Evaluation

**DOI:** 10.1093/ofid/ofab466.797

**Published:** 2021-12-04

**Authors:** Kalpana D Shere-Wolfe, Rachel Silk, Carla Alexander

**Affiliations:** University of Maryland, Baltimore, MD

## Abstract

**Background:**

Controversy and confusion surround the terminology for patients who have persistent symptoms after treatment for Lyme disease (LD) or may have been misdiagnosed with Lyme disease. While Infectious Diseases (ID) use the term Post treatment Lyme disease syndrome (PTLDS), patients tend to use the term Chronic Lyme disease (CLD) to describe the syndrome associated with persistent symptoms post treatment of LD. Many ID physicians are reluctant to see patients who identify themselves as having “Chronic Lyme” disease in some part due to reluctance to prescribe repeated courses of antibiotics. The purpose of this inquiry was to assess belief regarding Lyme disease and treatment.

**Methods:**

Patients at the Integrated Lyme Program at the University of Maryland completed clinical intake forms which included questions on their familiarity and beliefs surrounding Lyme disease.

**Results:**

We evaluated 146 patient records from our Lyme Program Registry which began in December 2018. There were 57 (34.5%) males and 108 (65.5%)females with mean age of 51 years. Forty seven percentage of patients were referred by a physician and 53 % were self-referred. Approximately 50% (71/146) were treated with less 30 days of antibiotics, 37% (54/146) were treated with 1-6 months of antibiotics and 11.6% (17/146) were treated with >6months of antibiotics prior to their initial evaluation in our Lyme program. Sixty eight percentage of patients were familiar with the term CLD but only 44% percentage were familiar with term PTLDS. Approximately half of the patients ( 52%) believed that they currently had Lyme disease and 63% believed that their current symptoms were due to Lyme disease. Despite this only 18% believed that they needed antibiotics for Lyme disease at the time completing the form.

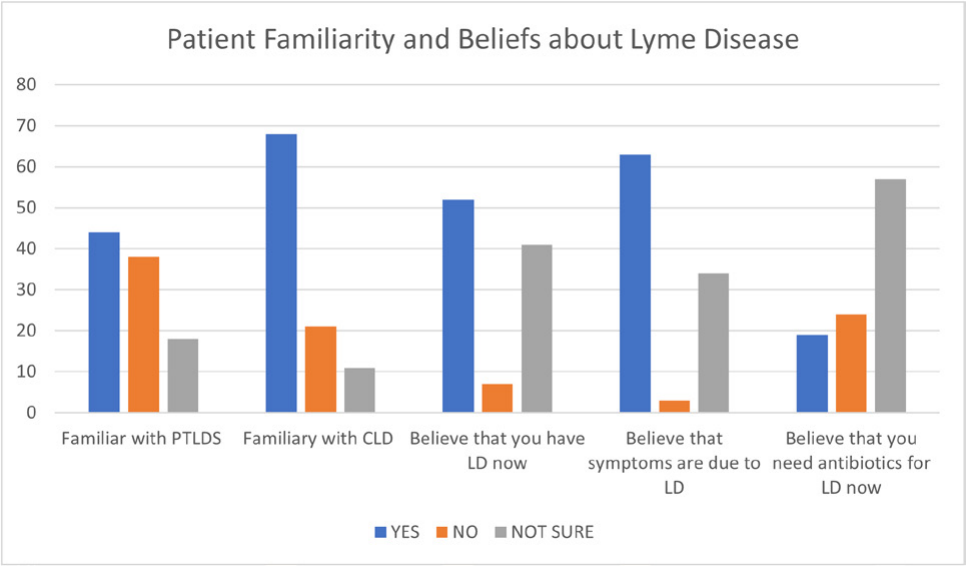

**Conclusion:**

Patient referred to our Lyme center were more familiar with term CLD vs PTLDS. Many of them believed that they currently had LD and their symptoms were due to Lyme disease. Despite this, the majority did not feel that they needed antibiotics for Lyme Disease at the time of their clinical visit. More research is needed to better understand patient beliefs and understanding regarding Lyme disease.

**Disclosures:**

**All Authors**: No reported disclosures

